# Motile Cilia on Kidney Proximal Tubular Epithelial Cells Are Associated With Tubular Injury and Interstitial Fibrosis

**DOI:** 10.3389/fcell.2022.765887

**Published:** 2022-03-14

**Authors:** Jennifer Eymael, Brigith Willemsen, Joyce Xu, Fieke Mooren, Eric Steenbergen, Jack F. Wetzels, Henry Dijkman, Jitske Jansen, Johan Van der Vlag, Bart Smeets

**Affiliations:** ^1^ Department of Pathology, Radboud Institute for Molecular Life Sciences, Radboud University Medical Center, Nijmegen, Netherlands; ^2^ Department of Nephrology, Radboud Institute for Health Sciences, Radboud University Medical Center, Nijmegen, Netherlands; ^3^ Department of Paediatric Nephrology, Radboud Institute for Molecular Life Sciences, Amalia Children’s Hospital, Radboud University Medical Center, Nijmegen, Netherlands; ^4^ Department of Nephrology, Radboud Institute for Molecular Life Sciences, Radboud University Medical Center, Nijmegen, Netherlands

**Keywords:** motile cilia, multiciliated cells, multiciliated differentiation, kidney injury and repair, interstitial fibrosis and tubular atrophy, proximal tubular cells, dedifferentiated

## Abstract

It is well established that mammalian kidney epithelial cells contain a single non-motile primary cilium (9 + 0 pattern). However, we noted the presence of multiple motile cilia with a central microtubular pair (9 + 2 pattern) in kidney biopsies of 11 patients with various kidney diseases, using transmission electron microscopy. Immunofluorescence staining revealed the expression of the motile cilia-specific markers Radial Spoke Head Protein 4 homolog A, Forkhead-box-protein J1 and Regulatory factor X3. Multiciliated cells were exclusively observed in proximal tubuli and a relative frequent observation in human kidney tissue: in 16.7% of biopsies with tubular injury and atrophy (3 of 18 tissues), in 17.6% of biopsies from patients with membranous nephropathy (3 of 17 tissues) and in 10% of the human kidney tissues derived from the unaffected pole after tumour nephrectomy (3 of 30 tissues). However, these particular tissues showed marked tubular injury and fibrosis. Further analysis showed a significant relation between the presence of multiciliated cells and an increased expression of alpha-smooth-muscle-actin (*p*-value < 0.01) and presence of Kidney-injury-molecule-1 (*p*-value < 0.01). Interestingly, multiciliated cells co-showed staining for the scattered tubular cell markers annexin A2, annexin A3, vimentin and phosphofructokinase platelet but not with cell senescence associated markers, like (p16) and degradation of lamin B. In conclusion, multiciliated proximal tubular cells with motile cilia were frequently observed in kidney biopsies and associated with tubular injury and interstitial fibrosis. These data suggest that proximal tubular cells are able to transdifferentiate into multiciliated cells.

## Graphical Abstract

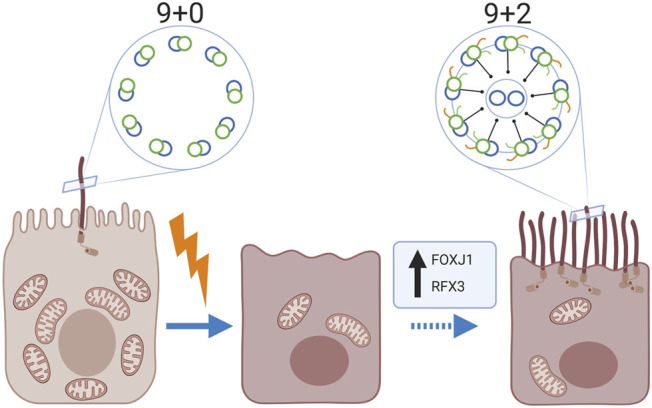

## Introduction

Cilia are evolutionary highly conserved antenna-like structures, which extend from the cell surface and consist of a highly organized array of microtubules. Two main types of cilia with different functionalities, the primary or sensory cilia and the motile cilia, can be distinguished by their microtubular organization. Primary cilia are composed of nine peripheral microtubule doublets but generally lack a central microtubule pair (9 + 0 structure). They extend as a single cilium from the cell surface in many tissues such as the kidney, the bile duct, the heart, the retina of the eye and on the olfactory neurons in the nose accomplishing functions as mechanosensors, chemosensors, and photosensors and are highly specialized organelles important for physiological processes such as cell signalling, embryonic development and wound healing (Yoder 2007; Shah et al., 2009).

Motile cilia are composed of nine peripheral microtubule doublets surrounding a central microtubule pair (9 + 2 structure). These cilia additionally express inner and outer dynein arms, radial spoke proteins and nexin links. Dynein arms are required for the generation of force, whereas radial spokes regulate the direction of ciliary beating and connect the peripheral to the central microtubular doublets which are connected to each other by nexin links (Cardenas-Rodriguez and Badano 2009). Ciliary beating is essential for motile cilia function. The formation of MCC and the assembly of motile cilia requires the activation of a transcriptional program in which Forkhead box protein J1 (FOXJ1) and Regulatory Factor X (RFX) proteins play essential roles (Choksi et al., 2014). Motile cilia can be found as monocilium (e.g. the flagellum of sperm cells) or combined on multiciliated cells (MCC) e.g. the respiratory tract epithelium and ependymal cells of the central nervous system of mammals. Motile cilia are designed to move fluid of high viscosity. This is important for the clearance of the airways, sinus and ears as well as the prevention of infections and inflammations by elimination of foreign particles in these tissues. Motile cilia are also important during the development since cells with a single motile cilium produce a fluid flow which establishes the left-right patterning in the early embryo (Bisgrove and Yost 2006). Motile ciliary dysfunction is a cause of infertility, airway diseases and hearing defects (Fliegauf et al., 2007; Plesec et al., 2008).

It is generally acknowledged that mammalian kidney epithelial cells contain a single non-motile primary cilium (9 + 0 pattern) at the apical side (Latta et al., 1961; Pfaller and Klima 1976), which extends into the tubular lumen and is believed to function as a mechanical sensor of urine flow (Yoder 2007; Praetorius and Leipziger 2013). Based on the sensed flow rate, the tubule diameter is regulated (Rodat-Despoix and Delmas 2009). Although, there are scattered published observations reporting the presence of MCC in diseased kidney (Duffy and Suzuki 1968; Katz and Morgan 1984; Lungarella et al., 1984; Hassan and Subramanyan 1995; Ong and Wagner 2005). The existence of MCCs in human kidney is not acknowledged and is thought to be an extremely rare event. Furthermore, no associations have been made so far between the presence of MCC and underlying pathology.

The origin of MCC in the kidney is unknown. Recent studies have shown that proximal tubular cells are plastic cells that in a response to injury can change their phenotype to become so called scattered proximal tubular cells. This cell population can be found in a scattered pattern in the proximal tubule and are possibly involved in the regeneration processes of the proximal tubule after tubular injury (Lindgren et al., 2011; Smeets et al., 2013; Berger et al., 2014). Furthermore, scattered tubular cells presumably represent a transient regenerative phenotype following a different transcriptional program compared to differentiated tubular epithelial cells. A possibility would be that also MCC derive from dedifferentiated proximal tubule cells because of a distinct activation of a transcriptional program.

In this study, we studied the presence of MCC in human kidneys and characterized the microtubular organization of the cilia found on MCC cells in the kidney as well as the cellular phenotype of the cells. Furthermore, it was assessed if the presence of MCC is associated with tubular injury or kidney fibrosis.

## Methods

### Patient Selection

All experimental protocols were approved by the ethical committee of the Radboudumc. The methods were carried out in accordance with the relevant guidelines and regulations. All patients provided written informed consent. Multiciliated tubular cells were found, by coincidence, during routine diagnostic procedures in kidney biopsies of 11 patients. Additionally, 18 patients were randomly selected based on the presence of tubulopathy, tubular atrophy and tubulointerstitial fibrosis and 17 patients were selected based on the diagnosis of membranous nephropathy. Additionally, 30 human kidney tissues, obtained from the unaffected pole from tumour nephrectomies were used. Archived kidney biopsies were selected with consent from the local ethics board of the Radboud university medical center (file numbers 2018–4563 and 2017–3652).

### Immunohistochemistry

Periodic acid-Schiff staining was performed on 3 µm thick sections as described before (Smeets et al., 2013).

For immunohistochemistry analysis, 4 µm thick paraffin embedded tissue sections were deparaffinised and rehydrated according to standard protocol. Antigen retrieval was performed in EDTA (pH 8.0). After the antigen retrieval, slices were blocked with 20% (v/v) normal horse serum. Endogenous biotin, biotin receptors, and avidin were blocked with the Avidin/Biotin blocking Kit (Vector laboratories SP-2001). Endogenous peroxidases were blocked with 0.3% (v/v) hydrogen peroxide in PBS. Tissues were then incubated with the first antibody, followed by an incubation with the secondary antibody (Tables 1, 2). VECTASTAIN®ABC-AP staining Kit (Vector laboratories, PK-5000) was applied followed by incubation of the StayBlue/AP solution (ab178453, abcam). After washing the slide thoroughly, the tissues were blocked again before incubating with the second primary antibody overnight at 4 C. Tissues were incubated with the secondary antibody and AEC substrate (ab64252, Abcam) was used as developing agent (red). The slides were mounted with Paramount and examined under a light microscope.

**TABLE 1:** Additional biopsies of patients selected on the presence of tubular injury and tubular atrophy.

Patient	Sex	Age	Multiple cilia	Histopathology	Diagnosis
12	M	31	Yes	Moderate nodular glomerulosclerosis. 50% interstitial fibrosis and tubular atrophy	Diabetic glomerulosclerosis
13	M	68	Yes	80% globally sclerotic glomeruli. 80% cortical fibrosis and tubular atrophy	Thrombotic microangiopathy
14	M	69	Yes	Nodular diabetic glomerulosclerosis. 70% interstitial fibrosis and tubular atrophy	Diabetic glomerulosclerosis
15	M	61	No	Nodular diabetic glomerulosclerosis. 50% interstitial fibrosis and tubular atrophy	Diabetic glomerulosclerosis
16	M	67	No	No glomerular aberrations. 30% interstitial fibrosis and tubular atrophy	FSGS
17	F	64	No	FSGS lesions. 30% fibrosis and tubular atrophy	Tubulo-interstitial Nephritis
18	M	77	No	50% global sclerotic glomeruli. 50% interstitial fibrosis and tubular atrophy	Tubulo-interstitial Nephritis, secondary FSGS
19	M	64	No	25–30% globally sclerotic glomeruli. 10–20% interstitial fibrosis and tubular atrophy	Suspected thin GBM nephropathy
20	M	24	No	Mesangial matrix expansion. 10% interstitial fibrosis and tubular atrophy	IgA nephropathy
21	M	64	No	No glomerular aberrations. 40% interstitial fibrosis and tubular atrophy	No classifying diagnosis
22	F	64	No	50% global sclerotic glomeruli. Mesangial PAS positive deposits. 50% interstitial fibrosis and tubular atrophy	AL-amyloidosis
23	M	43	No	Mesangiocapillary pattern of injury. 10–20% interstitial fibrosis and tubular atrophy	Membranoproliferative glomerulonephritis
24	F	36	No	20% globally sclerotic glomeruli. 50% interstitial fibrosis and tubular atrophy. Severe tubulopathy	Thrombotic microangiopathy
25	M	21	No	Moderate mesangial expansion and hypercellularity. 60% interstitial fibrosis and tubular atrophy	IgA nephropathy
26	M	48	No	75% globally sclerotic glomeruli. Diabetic changes. 70% interstitial fibrosis and tubular atrophy	Diabetic glomerulosclerosis
27	M	63	No	Some FSGS lesions. 30% interstitial fibrosis and tubular atrophy	Secondary FSGS
28	M	41	No	10% segmental sclerotic glomerular lesions. ±10% glomeruli with capillary necrosis and fibrin. 10% interstitial fibrosis and tubular atrophy	ANCA glomerulonephritis
29	M	74	No	20% globally sclerotic glomeruli. ±40% interstitial fibrosis and tubular atrophy	Thrombotic microangiopathy

**TABLE 2:** Primary antibodies.

Primary Antibody		Host species	Company
anti-acetylated alpha tubulin	Acαtub	mouse	Sigma Aldrich
FITC labeled lectin Lotus tetragonolobus agglutinin	LTL-FITC		Vectorlabs
anti-aquaporin-1	AQP1	rabbit	Alpha diagnostics
anti-Radial Spoke Head Protein 4 homolog	RSPH4A	rabbit	Sigma-Aldrich
anti-Forkhead-box-protein J1	FOXJ1	rabbit	Sigma-Aldrich
anti-regulatory factor X3	RFX3	rabbit	Sigma-Aldrich
anti-alpha-smooth-muscle-actin	αSMA	mouse	Abcam
Kidney Injury Molecule 1	KIM-1	mouse	R&D Systems
Annexin A2	ANXA2	mouse	Santa Cruz Biotechnology, Inc
Annexin A3	ANXA3	Rabbit	Sigma-Aldrich
Vimentin	VIM	mouse	Thermo Fisher Scientific
Phosphofructokinase, platelet	PFKP	mouse	OriGene Technologies Inc
cyclin-dependent kinase inhibitor 1	p21	mouse	Immunologic
Lamin B1	LMNB1	rabbit	Atlas antibodies

### Immunofluorescence Staining

For immunofluorescence, 3–4 µm thick paraffin-embedded tissue sections were deparaffinised and rehydrated. Antigen retrieval was performed using citrate buffer (pH 6.0). Slides were incubated with the primary antibodies overnight at 4°C (Table 2). Immunofluorescence labeling was achieved by incubation with the secondary antibody (Table 3). For nuclear staining, slides were mounted with DAPI-Fluoromount G (Cat. No. 0100–20, Southern Biotech, Birmingham, United States). Tissue was examined using High content fluorescent microscopy (Leica DMI6000B).

**TABLE 3:** Secondary antibodies.

Secondary Antibody	Company
Alexa donkey anti rabbit 568	Thermo Fisher Scientific
Alexa donkey anti mouse 488	Thermo Fisher Scientific
Alexa donkey anti mouse 647	Abcam
Alexa goat anti rabbit 568	Thermo Fisher Scientific
Horse anti mouse biotinylated	Vector laboratories
Poly-HRP-GAMs/Rabbit (ready to use)	Immunologic

### Tissue Analysis

Presence of MCC were manually determined using the High content fluorescent microscope based on multiciliated cell marker expression. KIM-1 was scored based on its presence in the tissue (Yes/No). The amount of interstitial fibrosis was based on the level of αSMA expression. The scoring was done using an automatic threshold for the αSMA expression and by calculating its surface area compared to the total surface area of the tissue in ImageJ ((Version 1.51). Not automated scoring was performed by two independent researchers.

### Transmission Electron Microscopy

For electron microscopy, kidney biopsies were fixed in 2.5% (v/v) glutaraldehyde dissolved in 0.1 M sodium cacodylate buffer, pH 7.4 for 24 h and washed in the same buffer. The biopsies were post-fixed with 2% (v/v) osmium tetroxide diluted in palade buffer for 1 h followed by dehydration in an increased series of ethanol followed by propylene oxide and embedded in Epon. Semi-thin sections were cut on an ultra-microtome (Reichert Ultracut S, Leica Microsystems, Amsterdam, Netherlands) and stained with toluidine blue. Ultra-thin sections were stained with 6% (v/v) uranyl acetate for 30 min and subsequently with lead citrate for 10 min at room temperature. Sections were examined in a JEOL 1400 electron microscope (JEOL, Tokyo, Japan); images were taken by Gatan Digital camera.

### Statistical Analysis

To test the statistical significance of the relation between the presence of MCC and the presence of KIM-1 expression in the same kidney tissue, a two-sided Fisher’s exact test was performed (statistically significant alpha <0.05). To determine the association between the amount of αSMA-expression and the presence of MCC, a two-tailed *t*-test (Mann-Whitney-*U* test) was performed and stated as significant with *p* < 0.05.

## Results

Multiciliated cells are present in the proximal tubules of patients with renal injury.

**FIGURE 1:**
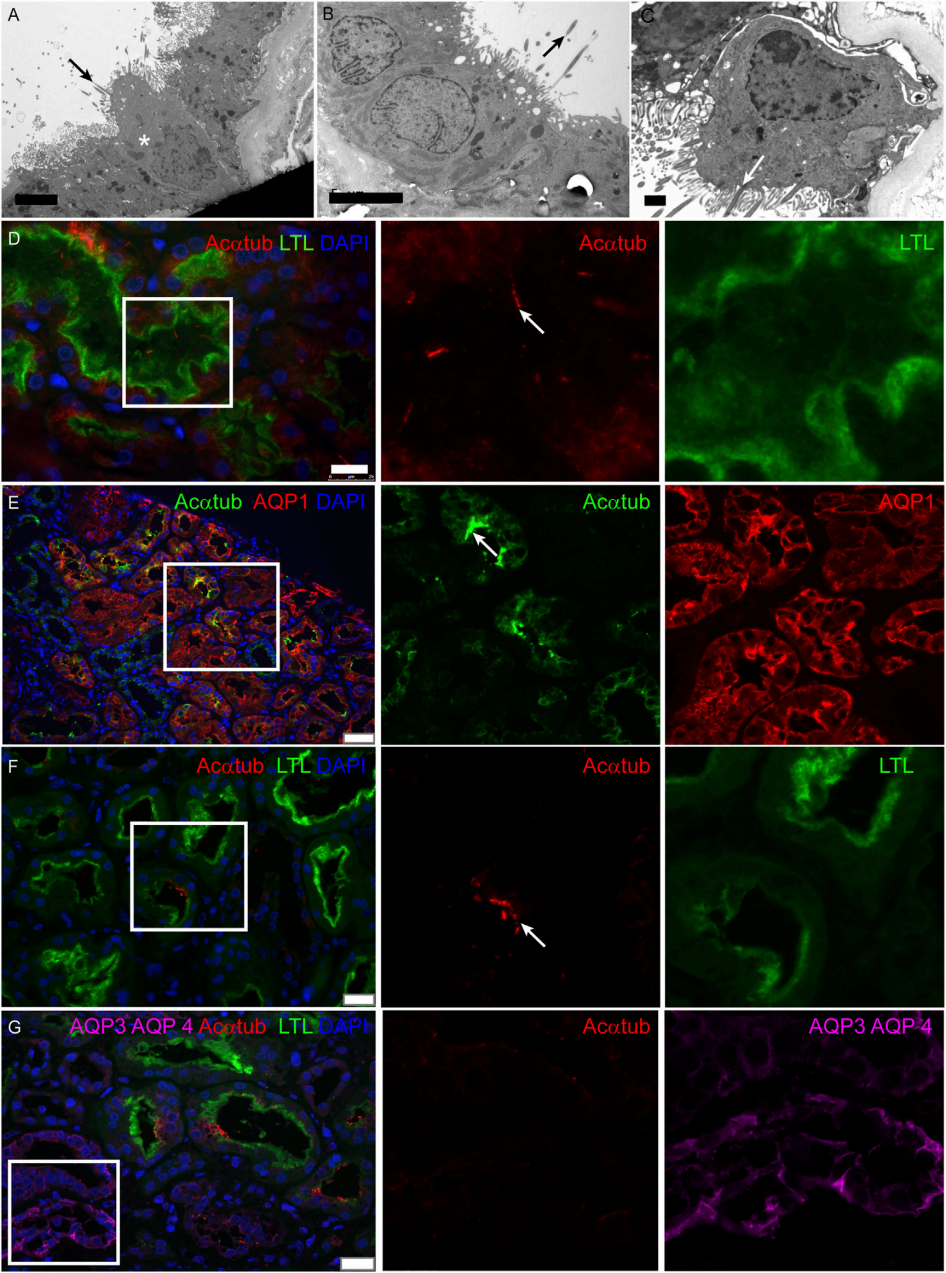
Multiciliated cells can be found in biopsies of patients with renal injury and are localized to the proximal tubule segment: **(A-C)** Representative images of MCC found during diagnostic analysis of kidney biopsies using transmission electron microscopy. Cilia extend into the tubular lumen (black arrows). Multiple cilia are anchored into the cell membrane of 1 cell (white arrow). Surrounding proximal tubular cells (asterisks) are characterized by an intact brush border and basal labyrinth, identifying them as proximal tubular cells. **(D)** Immunofluorescence staining of normal human kidney tissue for cilia detection using acetylated α-tubulin (Acαtub) (red). The section was co-stained for the proximal tubular brush border marker Lotus Tetragonolobus agglutinin Lectin (LTL) (green). **(E-G)** Immunofluorescence staining of kidney biopsies for specific tubular markers to localize multiciliated tubular cells using acetylated α-tubulin as cilia marker (E, green) (F-G, red). MCC present inside the proximal tubule indicated by **(E)** expression of aquaporin-1 (AQP1) (red) and **(F-G)** LTL (green) and **G)** not identified to be present in the collecting duct stained for Aquaporin 3 (AQP3) and Aquaporin 4 (AQP4) (purple, asterisks). Scale bars: **(A-B)** 5 μm, **(C)** 1 μm, **(D)** 25 μm**, (E)** 50 μm**, (F-G)** 25 µm.

Multiciliated cells were detected in 11 kidney biopsies during routine diagnostic transmission electron microscopy. The patient information and pathological findings are summarized in Table 4 (patient 1–11). Representative histological images of kidney tissue of the 11 patients in which MCCs were detected, are shown in Supplementary Figure S1. Interestingly, although all 11 patients had different underlying pathologies, they all showed tubular injury, tubular atrophy and tubulointerstitial fibrosis. In these biopsies multiple cilia, extending from proximal tubular cells and anchored to the cells by several basal bodies, could be detected (Figures 1A–C). To confirm the presence of MCC in these renal biopsies, immunofluorescence staining was performed on renal tissue of normal human kidney and on patient biopsies with MCC detected by transmission electron microscopy. Acetylated α-tubulin (Acαtub) and ADP-ribosylation factor-like protein 13B (ARL13B), a GTPase specifically localized in cilia, were used as cilia markers. In normal human kidney, singular cilia were detected (Figure 1D and Supplementary Figure). In patient kidney biopsies, we also detected single cilia, although their number seemed to be lower compared to the normal human kidney tissues. Importantly, the EM results were confirmed, as also in the immunofluorescence stainings MCC were detected (Figure 1E) with a strong variation in number between patients, ranging from few MCC in the entire biopsy to several MCC within a single tubular cross-section. Double staining with tubular segment-specific markers confirmed that MCC are only located in the proximal tubules and not in the distal segments. This was detected by co-localization of the cilia marker Acαtub limited to the proximal tubule specific marker Aquaporin-1 (AQP1) or Lotus tetragonolobus lectin (LTL), not co/localizing with distal tubule segment marker aquaporin-3 (AQP3) and aquaporin-4 (AQP4) (Figure 1E–G). Notably, transmission electron microscopy and immunofluorescence revealed a distinct phenotype of MCC. Compared to adjacent normal appearing mono-ciliated tubular cells, MCC showed a phenotype normally associated with proximal tubular epithelial cell injury and dedifferentiation. This dedifferentiated phenotype includes the loss of the basal labyrinth, fewer mitochondria, a rudimentary tubular brush border and an increased presence of deposits and vacuoles (Berger et al., 2014).

**TABLE 4:** Characteristics of patients with multiciliated cells detected during routine diagnostic examination of kidney biopsies.

Patient	Sex	Age	Multiple cilia	Histopathology	Diagnosis
1	F	29	Yes	A few FSGS lesions. Moderate interstitial fibrosis. Moderate tubulitis and tubular atrophy	Secondary FSGS
2	M	68	Yes	FSGS lesions. 30–40% intimafibrosis and tubular atrophy and minimal tubulopathy	Secondary FSGS
3	M	68	Yes	No glomerular aberrations. Tubulo-interstitial nephritis with moderate to severe tubulopathy	Tubulo-interstitial Nephritis
4	F	60	Yes	FSGS lesions. 40% interstitial fibrosis and tubular atrophy	IgA nephropathy
5	M	51	Yes	Nodular glomerulosclerosis. 70% interstitial fibrosis and tubular atrophy	Diabetic glomerulosclerosis
6	F	48	Yes	Thrombotic microangiopathy with FSGS lesions. Moderate interstitial fibrosis and tubular atrophy	Thrombotic microangiopathy with secondary FSGS
7	M	77	Yes	No glomerular aberrations. 50% interstitial fibrosis and tubular atrophy	Chronic tubulo-interstitial nephritis
8	M	74	Yes	50% glomerular sclerosis, 50% cortical fibrosis and tubular atrophy	Chronic damage in patient known with anti-GBM glomerulonephritis
9	M	38	Yes	FSGS lesions, approximately 2/3rd of the glomeruli. 50% interstitial fibrosis and tubular atrophy	FSGS NOS
10	M	55	Yes	Mild mesangial proliferation, in approximately 25% of the glomeruli segmental extracapillary proliferation. ±60% interstitial fibrosis and tubular atrophy	IgA nephropathy
11	M	68	Yes	FSGS lesions, approximately 1/3rd of the glomeruli. 60% interstitial fibrosis and tubular atrophy	No classifying diagnosis

### Multiciliated Proximal Tubular Cells Express Transcription Factors Associated With Motile Cilia Assembly and Ciliogenesis

**FIGURE 2:**
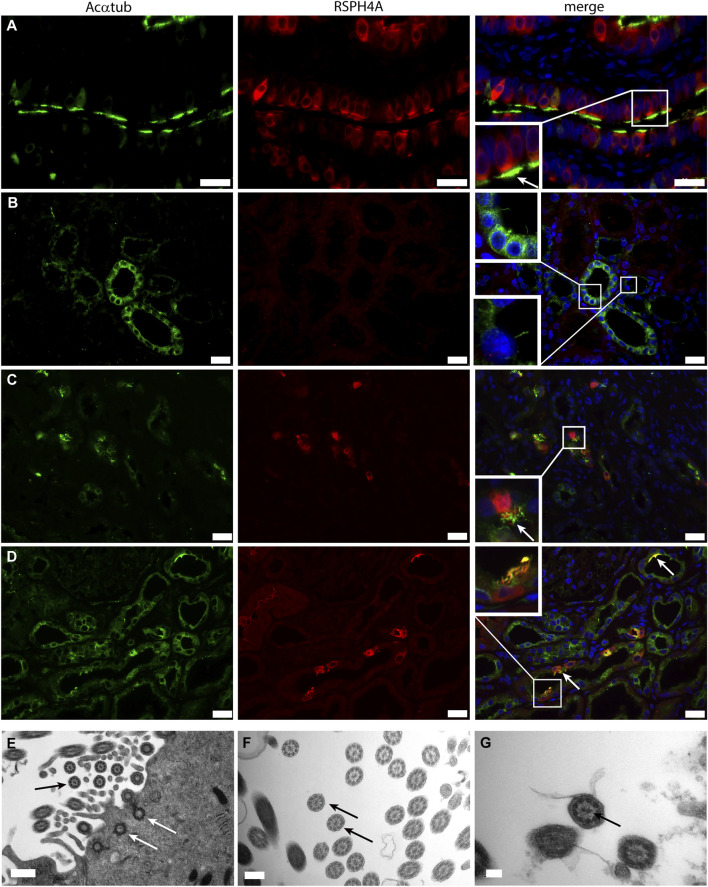
Motile cilia formation is present on renal multiciliated cells**: (A-C)** Immunofluorescence staining to distinguish between motile and non-motile cilia using acetylated α-tubulin (Acαtub) for cilia detection (green) and antibodies against the motile cilia marker Radial Spoke Head Protein 4 homolog A (RSPH4A) (red) in **(A)** Ovarian duct tissue used as a positive control, **(B)** Normal human kidney tissue, and **(C,D)** Renal biopsies of patients with tubular injury. RSPH4A was detected in the cilia as well as in the cell body of MCC of the ovarian duct and in patient kidney tissue but not in normal human kidney tissue. **(D-F)** Transmission electron microscopy of MCC present in renal biopsies showing multiple basal bodies anchored into 1 cell at the luminal side of the cell surface inside the cytoplasm (white arrows). The cilia ultrastructure is characterized by 9 peripheral microtubular doublets as well as a central microtubular pair (9 + 2 structure) (black arrows). Scale bars: **(A-C)** 25 μm, **(D)** 0.5 µm**, (E)** 200 nm, **(F)** 100 nm.

Next, we wanted to characterize the ciliary structure and questioned if the cilia present on multiciliated proximal tubular cells in patient biopsies were motile cilia, which are usually found on MCC in other organs (i.e., lung and ovary). To this end, the microtubular architecture and the expression of Radial Spoke Head Protein 4 homolog A (RSPH4A), which is only expressed in motile ciliated cells, was examined. All patient biopsies containing MCC detected in transmission electron microscopy showed cilia with a 9 + 2 structure. In addition, in the patient biopsies, RSPH4A was detected in MCC and was not present in normal human kidneys (Figure 2). The finding of a 9 + 2 structure using transmission electron microscopy is the strongest evidence for motile cilia. Every cell showing multiple cilia detected in the EM analysis was photographed and analysis of the images containing a high magnification of cross-sections of the cilia was performed (e.g. as depicted in the EM micrographs of Figures 2E–G). 94% of the MCC showed a 9 + 2 rearrangement, only in a few cells (approximately 6%) we could not detect a central microtubule pair. However, other MCC in the same biopsy MCC showed a motile cilium configuration. In summary, all patient biopsies containing MCC detected in transmission electron microscopy showed cilia with a 9 + 2 structure.

**FIGURE 3:**
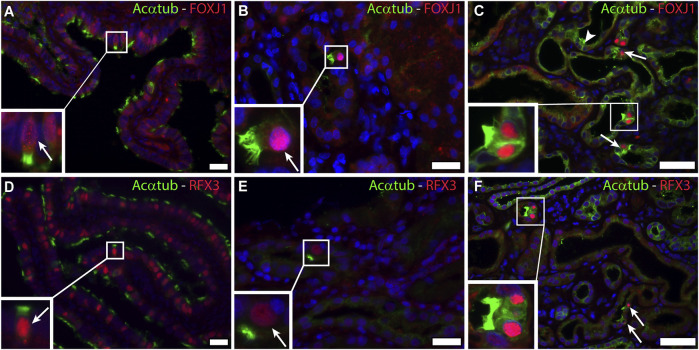
Activation of a common transcriptional program for motile cilia formation involving FOXJ1 and RFX3 is present in renal biopsies: Immunofluorescence staining for acetylated α-tubulin (Acαtub) as cilia marker (green) and the transcriptional factor Forkhead-box-protein J1 (FOXJ1) (red) in **(A,D)** Ovarian duct tissue as positive staining control and **(B,C)** Renal biopsies showing co-expression of FOXJ1 and acetylated α-tubulin in MCC. Immunofluorescence staining for acetylated α-tubulin as cilia marker (green) and the transcriptional factor Regulatory Factor X3 (RFX3) (red) in **(D)** Ovarian duct tissue as positive staining control and **(E,F)** Renal biopsies showing co-expression of RFX3 and acetylated α-tubulin in MCC present in renal biopsies. Scale bars: **(A,B,D,E)** 25 μm, **(C,F)** 50 µm.

The transcriptional program for motile cilia assembly and multicilia-formation involves transcriptional activation of FOXJ1 and the RFX family. Both transcription factors are essential for the formation of multiple basal bodies and the assembly of the motile cilia structure (Choksi et al., 2014). Staining of patient biopsies for FOXJ1 and RFX3 revealed nuclear expression of both transcription factors that was restricted to MCC (Figure 3). In normal human kidneys no expression of FOXJ1 and RFX3 was detected (data not shown).

### Multiciliated Cells are Frequently Present in Patients with Tubular Injury and Interstitial Fibrosis

**FIGURE 4:**
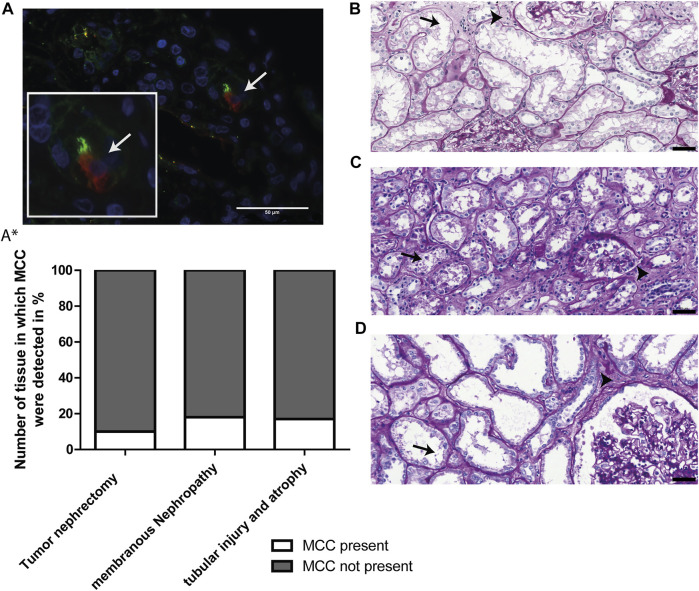
MCCs are frequently present in patients with renal injury. **(A)** Representative image of immunofluorescence staining for motile cilia marker RSPH4A (red) in combination with Acαtub (green) to detect MCC in renal tissue. (A*) Screening of Human Kidney tissue derived from the unaffected pole after tumour nephrectomy (Nephrectomy specimen), tissue of patients diagnosed with Membranous Nephropathy (MN) and patient biopsies selected for the presence of tubular injury and atrophy. The percentage of tissues in which MCC were (not) detected is shown. In 17.6% of patients with membranous nephropathy, 16.7% of patients with tubular injury and atrophy and 10% of Human Kidney tissue derived from the unaffected pole after tumour nephrectomy, MCC were found. **(B-D)** Representative images of PAS-staining of the three human kidney tissues derived from the unaffected pole after tumour nephrectomy in which MCC were found. In all tissues, marked proximal tubular cell injury (arrows) and tubulointerstitial fibrosis (arrowheads) could be detected. Scale bars: **(A-D)** 50 µm.

To study the significance of the aforementioned findings that MCC have been detected in kidney tissue showing tubular injury, tubular atrophy and tubulointerstitial fibrosis, we questioned how frequently MCCs can be found in human kidney and if this observation can be linked to kidney injury and regeneration. This was evaluated using immunofluorescence staining for the multiciliated cell marker RSPH4A (red) in combination with Acαtub (green) to detect MCC (white arrow, Figure 4A). The percentage of tissues that contained MCCs was assessed in 3 different groups of tissues. 1) 18 biopsies, from patients with different underlying kidney pathologies, that showed interstitial fibrosis and tubular atrophy (IFTA). The patient information of those biopsies can be found in Table 1. 2) 17 biopsies of patients with the same etiology i.e. membranous nephropathy, with various degrees of IFTA. The findings of the biopsies were compared to that of 30 additional human kidney tissues derived from the unaffected pole of the kidney after tumour nephrectomy. Tissue analysis revealed the presence of MCC in 3 of the 18 patients selected for tubular injury and atrophy (16.7%), in 3 of the 17 patients with membranous nephropathy (17.6%). Quantification of the percentage of proximal tubular expressing the MCC marker RSPH4A, in latter biopsies of patients with membranous nephropathy, revealed that 0,4 ± 0.1% of all proximal tubular cells in these biopsies expressed RSPH4A. Next to the diseased kidneys, MCC were also detected in 3 of 30 human kidney tissues derived from the unaffected pole of the kidney after tumour nephrectomy (10%) (Figure 4A*). Of note, analysis of the periodic-acid-Schiff (PAS) staining of those 3 kidney tissues, in which MCC were detected revealed tubular injury and interstitial fibrosis (Figures 4B–D). This indicates that these kidneys cannot be classified as physiologically healthy human kidneys. Taken together, analysis of the percentage of kidney tissues containing MCC revealed that assembly of multiple motile cilia is not a rare event in patients with renal injury.

Next, we assessed the association between the presence of MCC and proximal tubular cells injury and interstitial fibrosis.

**FIGURE 5:**
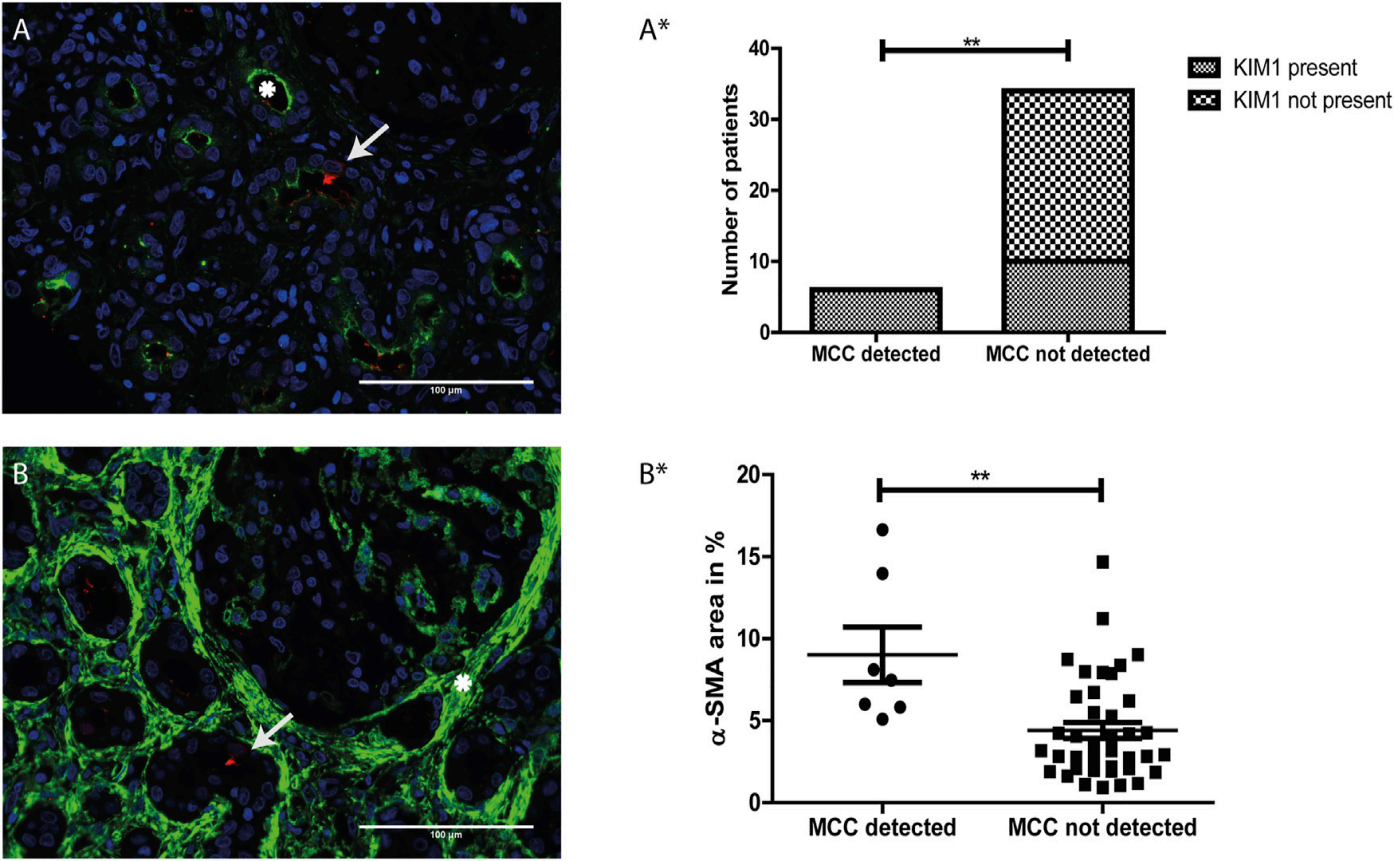
The presence of multiciliated cells is related to tubular injury and interstitial fibrosis: **(A)** Immunofluorescence staining for Kidney injury molecule 1 (KIM-1) (green) showing expression of KIM-1 located at the apical side of the proximal tubule (asterisk). Co-staining with RSPH4A (red) showed presence of a multiciliated cell in the same tissue (arrow). (A*) Analysis of the relation between the presence of MCC and KIM-1 expression. A two-sided Fisher’s exact test revealed a significant association of the presence of MCC and KIM-1, *p*-value = 0.0021 (**). **(B)** Immunofluorescence staining for aSMA expression (green), which is located in the interstitial space (asterisk). Co-staining with RSPH4A (red) showed the presence of a multiciliated cell in this area (arrow). (B*) Analysis of the relation between aSMA expression (calculated percentage of the surface area of the tissue showing aSMA signal) and the presence of MCC. A two-tailed *t*-test revealed a significant increase of αSMA expression in tissues in which MCC were detected, *p*-value = 0.0068 (**). Scale bars: **(A-B)** 100 µm.

Proximal tubular cell injury was determined by immunostaining for KIM-1 (green, white asterisks), a marker for proximal tubule injury (Figure 5A). Analysis revealed a significant association between the presence of MCC and KIM-1 expression (*p* value <0.01) (Figure 5A*).

Next, αSMA was used as a marker for interstitial fibrosis which is related to chronic kidney injury. The tissues were stained for αSMA and the percentage of αSMA positive surface area within the tissues was measured using automated analysis in ImageJ. In Figures 5A,B representative image of immunofluorescence staining with strong αSMA (green) staining in the interstitial space (white asterisk) is shown. Using a two-tailed *t*-test, analysis revealed a significant increase of αSMA expression in tissues in which MCC were detected (*p*-value < 0.01) (Figure 5B*).

In conclusion, MCC were absent in kidney tissue that were negative for KIM-1 immunostaining or showed less than 5% αSMA staining. Furthermore, MCC were present in some but not all kidney tissues stained positive for KIM-1. These findings suggest that the presence of MCC in the proximal tubule could be associated with proximal tubule injury and interstitial fibrosis.

### Multiciliated Cells are not Senescent Cells

**FIGURE 6:**
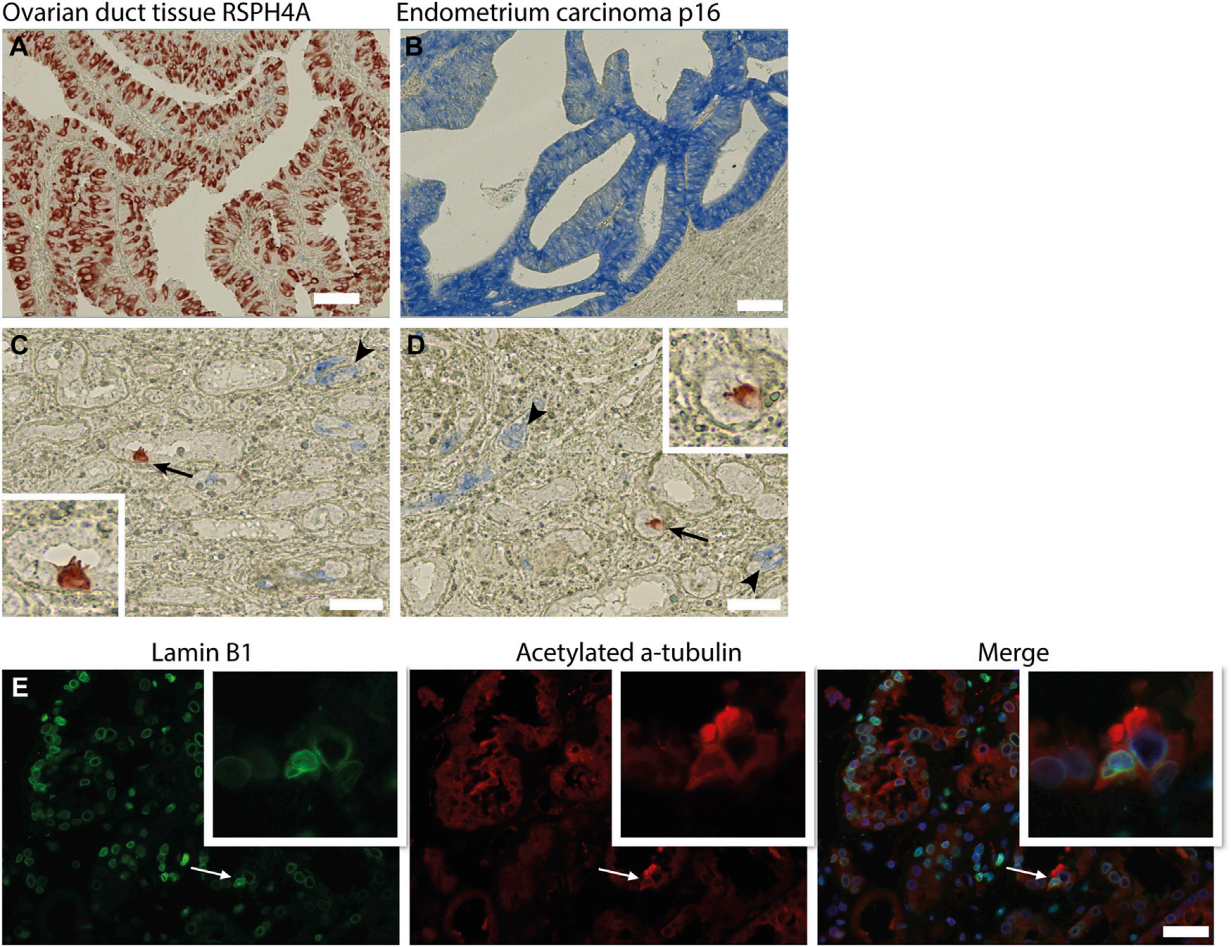
Multiciliated cells do not express markers associated with cellular senescence. (A) positive control tissue stained for RSPH4A and **(B)** p16. **(C,D)** Immunohistochemical double staining for RSPH4A as multiciliated cell marker (red) and senescence associated marker p16 (blue). RSPH4A stains single MCC (arrows and inset), p16 immunostaining resulted in bleu staining of epithelial cells in glomerular epithelial lesions as well in several small (atrophic) tubular structures (arrowheads). No co-staining of RSPH4A andp16 was detected. **(E)** Immunofluorescence staining for Lamin B1 and acetylated a-tubulin showing a multiciliated cell expressing Lamin B1 around the nucleus. Scale bars: **(A-E)** 50 µm.

In the kidney, cellular senescence has been associated with impaired regeneration of the tubular epithelium and tubular atrophy (Yang et al., 2010; Zhou et al., 2020). Furthermore, recent studies have shown that senescent cells are actively involved in kidney fibrosis mainly by secretion of senescence-associated secretory phenotype (SASP) components (Zhou et al., 2020). As the presence of MCCs was associated with kidney fibrosis, we questioned whether MCCs in the kidney are senescent. To test if MCC are possibly in a senescent state we performed immunohistochemical staining for the senescence markers p16 and lamin B1. The senescence marker p16 is a tumour suppressor gene that is activated by different factors including DNA damage and oxidative stress and is associated with cell cycle dysregulation in senescence (Beausejour et al., 2003; Ben-Porath and Weinberg 2005; Rayess et al., 2012). Cellular senescence is associated with the degradation of Lamin B1, which is a structural component of the nuclear lamina (Freund et al., 2012). Multiciliated cells identified by the expression of RSPH4A (red) did not show co-localization with p16 (blue) (Figures 6C,D). To determine if MCC show Lamin B1 degradation, immunofluorescence staining was performed for Lamin B1 together with acetylated α tubulin to detect MCC (Figure 6E). No loss of Lamin B1 around the nucleus of MCC was detected. In summary, MCC did not show a senescent phenotype.

### Multiciliated Cells Express Markers Associated With the Scattered Tubular Cell Phenotype

**FIGURE 7:**
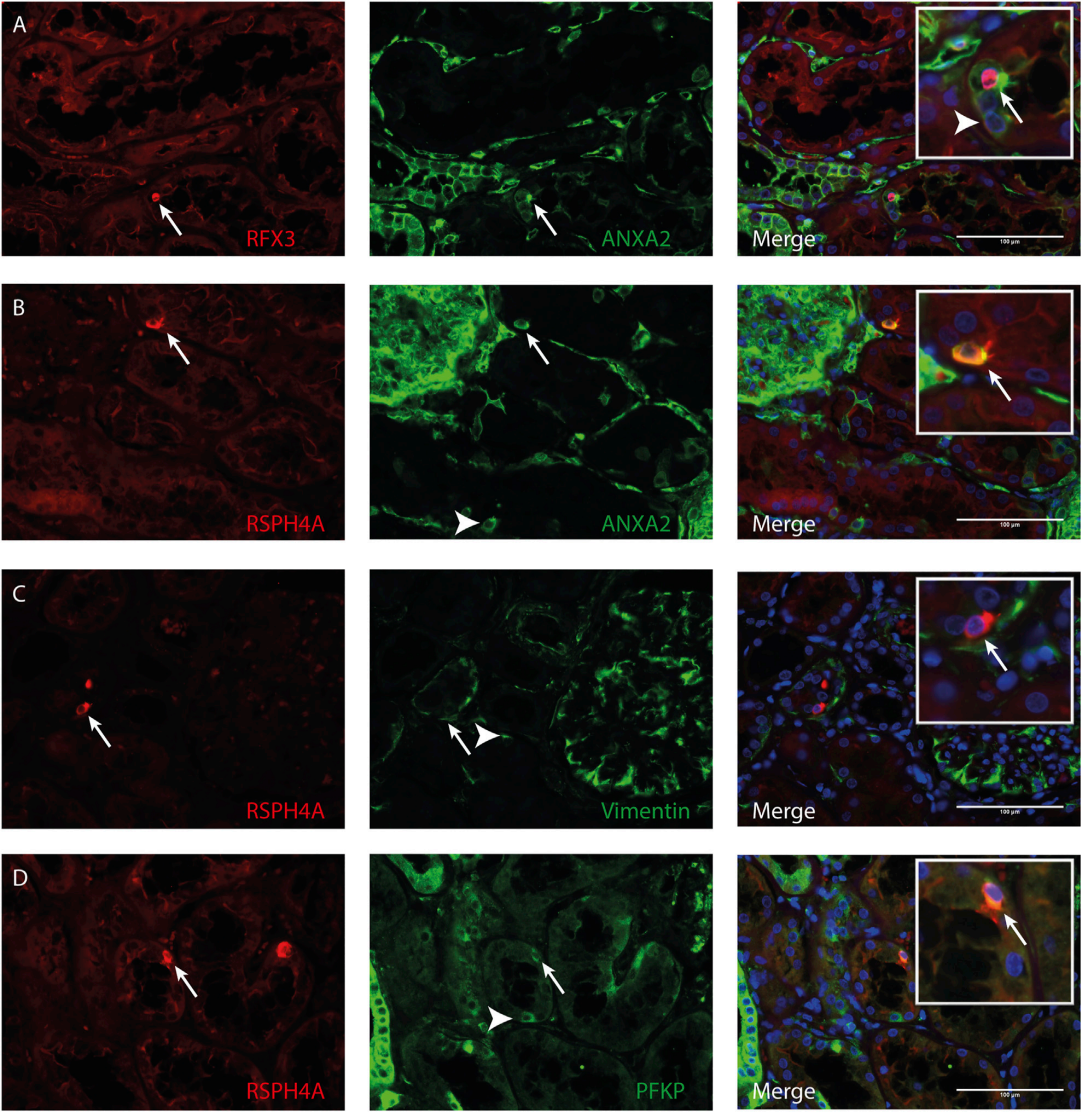
Co-localization of multiciliated cell marker with scattered tubular cell marker. Representative image of immunofluorescence staining using **(A)** RFX3 as multiciliated cell marker (red) together with Annexin A2 (ANXA2) as scattered tubular cell marker (green) showing co-localization in MCC (arrow). Representative images of immunofluorescence staining using RSPH4A as marker for motile cilia present in MCC (red) shows co-localization with scattered tubular cell markers (green) **(B)** Annexin A2 **(C)** Vimentin and **(D)** PFKP (arrows). Interestingly, in all tissues, also cells expressing scattered tubular cell markers can be observed which show no co-expression of RFX3 or RSPH4A (white arrowheads). Scale bars: **(A-D)** 100 µm.

**FIGURE 8:**
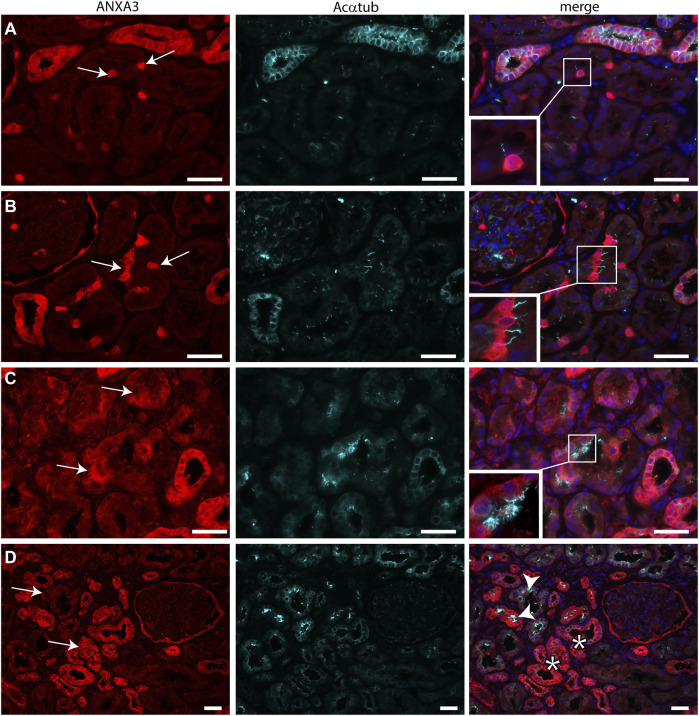
Scattered tubular cells can have a single cilium or multiple cilia. **(A,B)** normal human kidneys sections stained for the STC marker ANXA3 (red) and Acαtub (cyan). ANXA3 positive STC (arrows) in normal kidney tissue section contain a single Acαtub positive cilium. **(C,D)** Biopsy tissue sections showing a marked increase in number of STC marker positive tubular cells, entire tubular segments are positive for ANXA3 (arrows). Some ANXA3 positive cells show multiple Acαtub positive cilia C, insets, D, arrowhead). However, many ANXA3 positive tubular segments do not contain MCC **(D**, asterisks**)**.

Transmission electron microscopy showed that MCC had signs of dedifferentiation which are associated with tubular injury. A similar dedifferentiated phenotype was described for scattered tubular cells (STC), which are thought to be dedifferentiated proximal tubule cells which possibly play a role in tubular regeneration (Smeets et al., 2013). To analyse if MCC could originate from scattered tubular cells, immunofluorescence staining was performed for the multiciliated cell markers RSPH4A and RFX3 (red) together with scattered tubular cell markers Annexin A2, Vimentin and PFKP (green). We observed that cells expressing the multiciliated cell markers also express the STC markers (Figure 7). This finding was confirmed by co-stainings for the scattered cell marker ANXA3 and acetylated α tubulin. STC can also be detected in normal kidney tissue. The ANXA3 positive tubular cells contained single acetylated α tubulin positive cilia (Figures 8A,B). In the patient biopsies, there is a striking increase in the number of STC and some of these cells show an acetylated α tubulin positive MCC phenotype (Figures 8C,D). Additionally, Supplementary Video S1 shows confocal microscopy of a thick tissue (10 µm) with a single multiciliated cell expressing motile cilia marker RSPH4A and scattered tubular cell marker vimentin. However, MCCs represented only a small subpopulation of STC, as the vast majority of cells expressing scattered tubular cells markers were negative for the MCC markers. These results suggest that scattered tubular cells can become MCC.

## Discussion

It has been generally accepted that the healthy human kidney contains only primary sensory cilia. In this study, we show that multiciliated proximal tubule cells can be present in human kidney. Furthermore, cilia found on MCC were identified as motile cilia because of their 9 + 2 structure, expression of RSPH4A and the motile cilia assembly transcription factors FOXJ1 and RFX3, which are normally expressed in the lung, nasopharynx and ovarian epithelial cells. Our findings suggest the capability for ciliary motility in the proximal tubules of patients with tubular injury. Although, we observed the multiciliated proximal tubular cells in kidneys with areas characterized by tubular cell injury, there was no clear association with the underlying kidney disease. These observations suggest that a common pathological mechanism may trigger MCC transdifferentiation leading to motile cilia assembly in the damaged proximal tubule cells (Graphical abstract). Nevertheless, although we were able to detect MCC in ∼17% of the biopsies showing IFTA, in the majority of the biopsies with marked IFTA no MCCs were detected, suggesting that other or additional stimuli are necessary for motile multi-cilia assembly. Besides the striking finding of MCC, we generally observed fewer cilia in the diseased kidney. This observation fits with earlier publications showing that injury of the kidney epithelial cells can lead to shortening and loss of cilia (Park 2018; Fujii et al., 2021) There are other studies that reported the presence of MCC in diseased kidney (Duffy and Suzuki 1968; Katz and Morgan 1984; Lungarella et al., 1984; Hassan and Subramanyan 1995; Ong and Wagner 2005). In these studies, patients suffered from different pathologies (i.e. hypercalcemia, amyloidosis, lipoid nephrosis, membranoproliferative glomerulonephritis, focal segmental glomerulosclerosis and crescentic glomerulonephritis). As studies on MCC in the human kidney are scarce, the existence of these MCC has not been acknowledged and is thought to be an extremely rare event. Interestingly, in our study, this phenomenon was not a rare observation as we observed MCC in 16.7% of the randomly examined patients with tubular pathology as well as in 18% of examined membranous nephropathy patients. MCCs were also detected in 10% of the kidney tissues derived from the unaffected pole after tumour nephrectomy. Here, further tissue analysis revealed that those “normal” human kidneys in which MCC were found did not show a complete healthy morphology as we observed tubular injury, atrophy and interstitial fibrosis. In addition, the area of tissue analysed in the nephrectomies was much larger than the area analysed in biopsies from patients. Therefore, the percentages of MCC positive tissues cannot be compared and is likely to be overestimated in normal human kidney. We identified a significant association between the existence of MCC with motile cilia in the kidney and the presence of tubular injury and interstitial fibrosis identified by increased expression of KIM-1 and aSMA, respectively. However, to identify specific trigger(s) for the formation of MCC, an even larger patient cohort will be needed to allow study of the associations between patient characteristics, pathology, treatment and the presence of MCC.

The possible function of the MCC and underlying mechanisms leading to the formation and assembly of MCC remains unclear. In other organs like the nasopharynx and lung, motile cilia are physiologically present on multiciliated epithelial cells and are designed to move fluid of high viscosity. Interestingly, motile cilia inside the kidneys are evolutionary highly conserved as they can be found inside the excretory system of invertebrates and lower vertebrates contributing to solute and water movement along the renal structure. In the zebrafish kidney, the pronephric ducts contain mono-as well as multi-ciliated cells. These cilia were shown to be motile as they beat to create fluid movement (Kramer-Zucker et al., 2005). Loss of cilia motility and function leads to fluid accumulation resulting in organ swelling (Kramer-Zucker et al., 2005). Also, in organisms with a lower filtration pressure in the kidney such as amphibians, MCC are present in the proximal tubules, specifically in the neck segment, in which ciliary action compensates for the low pressure, facilitating flow from the glomerulus towards the tubules. One could speculate that assembly of multiple motile cilia in renal patients is an attempt to maintain tubular flow and/or clearance of the renal tubules from cell debris and could help to establish tubular flow and function in those cases with a reduced glomerular filtration and/or tubular obstruction (Uchida and Endou 1988; Khwaja 2012; Emma et al., 2016).

The question remains which events trigger the formation of these MCC and whether this is an adaptive response to injury or a maladaptive transcriptional activation of (dedifferentiated) injured proximal tubular cells. In recent studies, it was shown that tubular atrophy and interstitial fibrosis is associated with growth arrest and senescence of tubular epithelial cells. We examined if MCCs in the kidney are senescent and therefore possibly involved in fibrosis and impaired regeneration. However, no co-localization was found with the tested and well-known senescence marker, indicating that MCCs are not in a senescent state (Beausejour et al., 2003; Ben-Porath and Weinberg 2005; Rayess et al., 2012).

It is well known that the proximal tubule has a high capacity to regenerate after injury. Recent studies, using techniques like lineage tracing showed that proximal tubular cells are highly plastic and can dedifferentiate to acquire a phenotype that is less sensitive to injury and capable to proliferate (Smeets et al., 2013; Berger et al., 2014; Hansson et al., 2014; Kusaba et al., 2014; Eymael and Smeets 2016). This dedifferentiated phenotype is known as scattered tubular cell phenotype. We have found that cells that showed expression for a multiciliated cell maker, showed co-expression with a scattered tubular cell markers. However, not every scattered tubular cell showed co-expression for the multiciliated cell marker. Based on this observation, it can be assumed that the multiciliated cell population is possible a subpopulation of the scattered tubular cell population. Most importantly, the observations in this study indicate that dedifferentiated proximal tubular cells known as scattered tubular cell phenotype are capable to transdifferentiate into MCC. However, the signal which may induce the scattered tubular cell to multiciliated cell transition is still unknown.

In conclusion, proximal tubular cells with multiple motile cilia are frequently observed in patients with tubular injury indicating that proximal tubular cells are able to transdifferentiate into MCC. The mechanism and possible function underlying this phenomenon need further investigation.

## Data Availability

The original contributions presented in the study are included in the article/Supplementary Material, further inquiries can be directed to the corresponding author.
