# Supplementary figures and images for: Motile Cilia on Kidney Proximal Tubular Epithelial Cells Are Associated With Tubular Injury and Interstitial Fibrosis
Source: Front Cell Dev Biol. 2022 Mar 14;10:765887. doi: 10.3389/fcell.2022.765887 (PMC8964404; doi:10.3389/fcell.2022.765887)

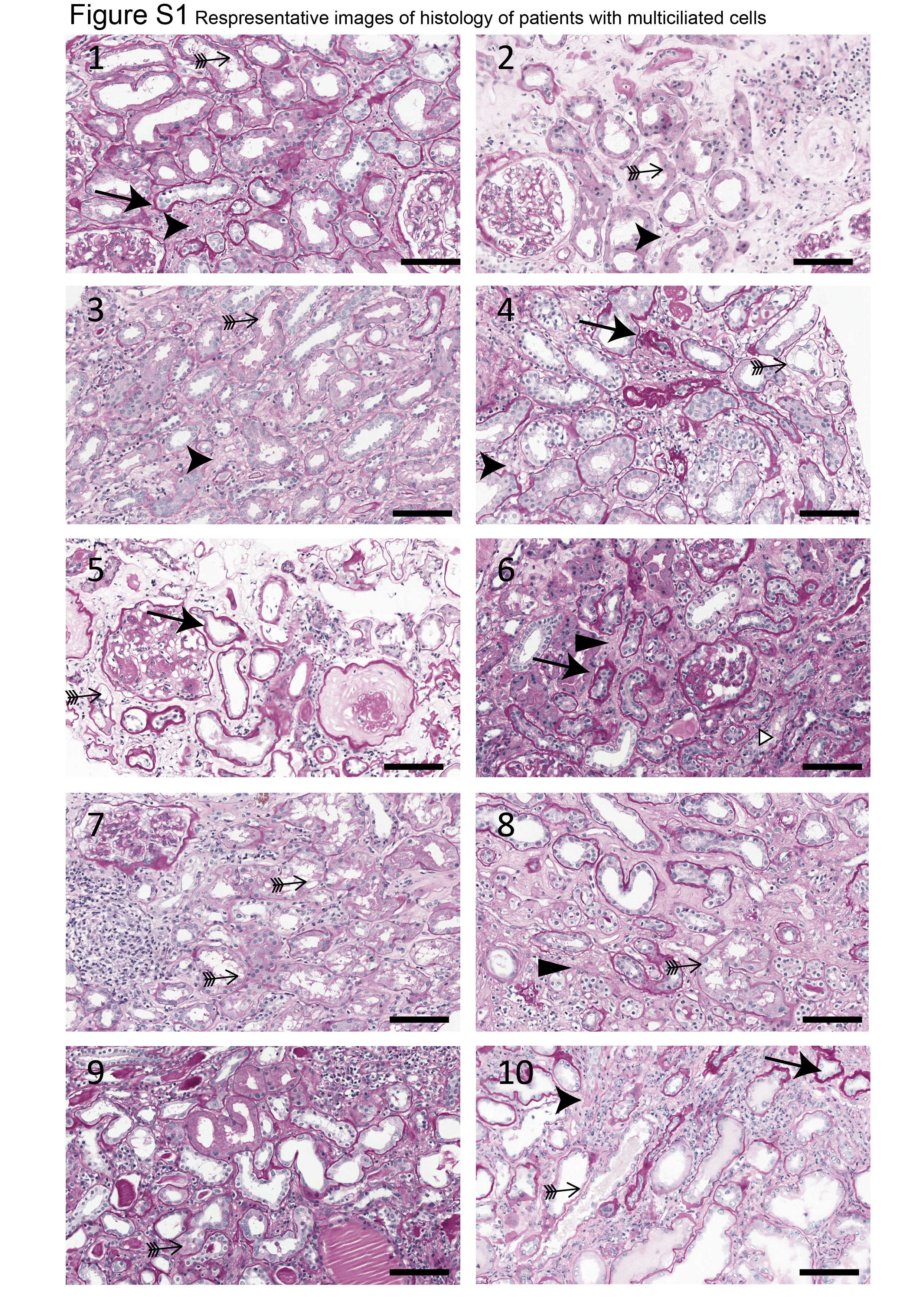

Supplement: Supplementary file 1 [file Figure10.JPEG]

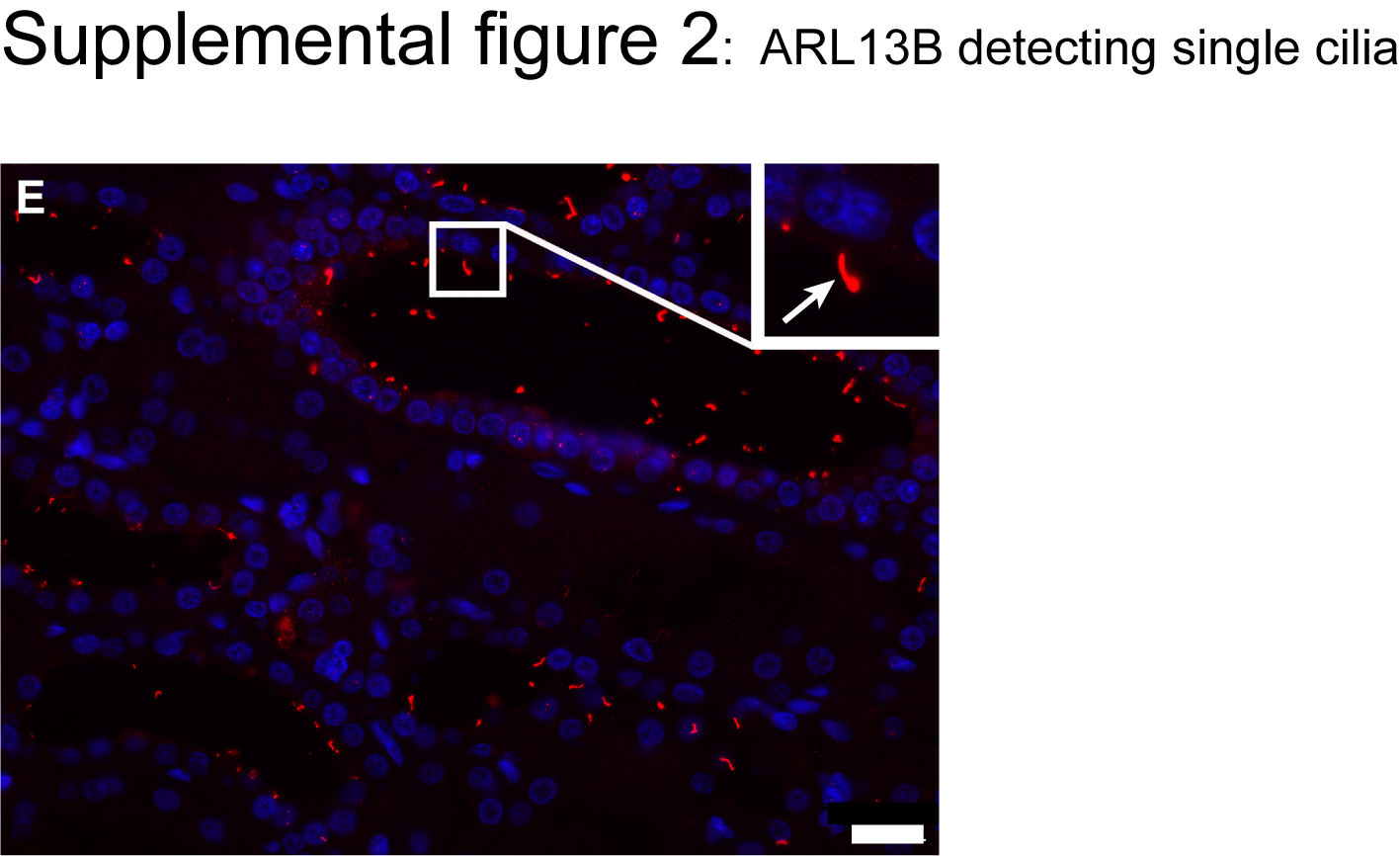

Supplement: Supplementary file 2 [file Figure11.TIF]
